# CYP450 Metabolizer Phenotypes in a Turkish Emergency Cardiac Patient Cohort: A Descriptive Pharmacogenomic Study

**DOI:** 10.3390/ph19060812

**Published:** 2026-05-22

**Authors:** Alten Oskay, Tülay Oskay, Veli Kaan Aydın, Özer Eser, Murat Seyit, Işık Tekin, Mert Özen, Atakan Yılmaz, İbrahim Türkçüer, Gergana Lengerova, Martina Bozhkova, Steliyan Petrov, Aylin Köseler

**Affiliations:** 1 Department of Emergency Medicine, Pamukkale University Faculty of Medicine, 20070 Denizli, Türkiye; aoskay@pau.edu.tr (A.O.); mseyit@pau.edu.tr (M.S.); mert@pau.edu.tr (M.Ö.); atakany@pau.edu.tr (A.Y.); iturkcuer@pau.edu.tr (İ.T.); 2 Department of Cardiology, Denizli State Hospital, 20000 Denizli, Türkiye; oskaytulay@gmail.com (T.O.); drozereser@hotmail.com (Ö.E.); 3 Department of Biophysics, Pamukkale University Faculty of Medicine, 20070 Denizli, Türkiye; vkaydin@pau.edu.tr; 4 Department of Cardiology, Pamukkale University Faculty of Medicine, 20070 Denizli, Türkiye; itekin@pau.edu.tr; 5 Department of Medical Microbiology and Immunology “Prof. Dr. Elissay Yanev”, Medical University of Plovdiv, 4002 Plovdiv, Bulgaria; gergana.lengerova@mu-plovdiv.bg (G.L.); martina.bozhkova@mu-plovdiv.bg (M.B.); steliyan.petrov@mu-plovdiv.bg (S.P.); 6 Research Institute, Medical University of Plovdiv, 4002 Plovdiv, Bulgaria; 7 Center of Competence—Personalized Innovative Medicine, 4002 Plovdiv, Bulgaria

**Keywords:** CYP3A4, CYP2D6, CYP2C19, polymorphisms, pharmacogenetics, cardiac patients

## Abstract

**Background/Objectives**: Cytochrome P450 enzymes (CYP2D6, CYP2C19, CYP3A4) play a key role in interindividual variability in cardiovascular drug metabolism. This study aimed to describe metabolizer phenotype distributions in a Turkish emergency cardiac cohort and across diagnostic categories. **Methods**: This retrospective descriptive pharmacogenomic study included 250 patients. Genotyping was performed using TaqMan assays for CYP2D6 (*2, *4, *10, *41), CYP2C19 (*2, *17), and CYP3A4 (*22, *1B). Phenotypes were assigned according to CPIC guidelines. CYP2D6 copy-number variation was not assessed. **Results**: Non-normal metabolizer phenotypes were observed in 55.6% (CYP2D6), 84.4% (CYP2C19), and 30.4% (CYP3A4) of patients. For CYP2D6, normal (44.4%) and intermediate (42.0%) metabolizers predominated. For CYP2C19, intermediate metabolizers were most frequent (36.0%), followed by normal (22.8%), rapid (17.2%), poor (14.8%), and ultra-rapid metabolizers (9.2%). CYP3A4 showed predominantly normal activity (69.6%). Phenotype distributions varied across diagnoses without clear clustering. **Conclusions**: A high prevalence of CYP2D6 and CYP2C19 variability with predicted functional relevance based on CPIC was observed, whereas CYP3A4 activity was more stable. These findings provide descriptive pharmacogenomic data to support future genotype-guided cardiovascular therapy studies.

## 1. Introduction

The genetic polymorphisms in the cytochrome P450 (CYP450) enzymes CYP3A4, CYP2D6, and CYP2C19 are known to influence drug metabolism and pharmacokinetics, and these effects are the basis for variable drug response and adverse drug effects in patients with cardiac disease [1]. Genetic differences in alleles such as CYP2D6 with different allelic variants, CYP3A4*1B and 22, CYP2C92, and *3 have a significant impact on both the efficacy and side effects of many cardiovascular drugs such as anticoagulants, statins, antiplatelets, and antiarrhythmics [2,3]. For instance, genetic variants in CYP2C9 determine response to warfarin, and CYP2C19 genotypes affect the pharmacokinetics and safety of mavacamten [3]. Similarly, changes in the gene encoding the CYP2D6 enzyme may alter the metabolism of various drugs used in the treatment of cardiovascular diseases, affecting the rate at which the drug is activated or deactivated [4]. Due to the significant clinical implications of these genetic differences, personalized medicine techniques are needed to increase treatment success and reduce side effects in cardiac patients. To this end, pharmacogenomic testing of CYP450 genotypes can facilitate personalized drug selection and dosing, particularly for medications requiring close therapeutic monitoring [5]. Merging all that knowledge, it is very important to know how frequently these gene variants occur and what their effect is on different patient populations. The study of the effect of CYP450 polymorphisms on clopidogrel response in Moroccan patients revealed a possible therapeutic efficacy in acute coronary syndromes [6]. Detailed pharmacogenomic studies are necessary to understand the role of genetic variations that occur on a case-by-case basis in various cardiac diseases and cause differences in drug metabolism [6]. Additionally, studies in other understudied populations may reveal novel genomic variations and unique allele frequencies important for pharmacogenomics [4].

The most important contributions of pharmacogenomics to the field of cardiology include the genotyping of genes such as CYP2C9 and CYP2C19, which significantly reduce unintended drug side effects and thus improve patient outcomes through a more personalized treatment approach [5,7]. This personalized method, guided by pharmacogenetics, has proven therapeutic efficacy in cardiovascular treatments such as clopidogrel response and warfarin dosage [8,9]. Cardiovascular diseases remain among the leading causes of death worldwide, and therefore, precision therapy is crucial [10]. In addition, the use of cutting-edge genetic testing techniques such as genome-wide association studies and multi-omics analyses accelerates this precision by opening the way for a more detailed understanding of the sources of individual differences in drug responses [11]. Such understanding is especially critical in various populations, as different allele frequencies and genetic backgrounds may have a considerable impact on drug metabolism and response [4].

A pilot study in the UAE demonstrated the feasibility of pharmacogenomic testing and found very high frequencies of genetic variants in a diverse population that were not anticipated, pointing out that there is a need for local pharmacogenomic guidelines [4]. In a similar vein, the utility of pre-emptive genotyping for CYP2C19 in providing guidance for antiplatelet therapy in acute coronary syndrome patients has been shown; however, a substantial gap is pointed out by the authors regarding the lack of racial and ethnic diversity in the studies of the outcomes [12,13]. The resolution of this difference is vital to the creation of informed pharmacogenetic guidelines, which can serve as a reliable tool in drug response prediction in different populations worldwide [14]. Therefore, it is necessary to conduct more studies to have a wider demographic representation in pharmacogenomic studies so that precision medicine can be a source of benefits to all patients irrespective of their ancestry or place of residence [15,16,17].

The goal of using pharmacogenomics in clinical practice, especially in cardiology, is to reduce the occurrence of adverse drug reactions, i.e., those that account for a large proportion of hospitalizations and health care costs [17,18]. Genetic variations in the CYP450 enzyme family, such as CYP3A4, CYP2D6, and CYP2C9, are the main factors that cause these adverse drug reactions by changing drug metabolism and potency [19,20,21]. Genetic differences in CYP2C19, for example, may alter the way antiplatelet drugs such as clopidogrel are metabolized, thus increasing the chances of major adverse cardiovascular events happening in patients with poor metabolizer phenotypes [22]. It basically shows the importance of pharmacogenetic testing as a tool to help doctors decide on the best personalized treatment to both enhance the therapeutic effect and reduce the risk of drug-related side effects in patients suffering from cardiac diseases [23,24].

Pharmacogenomics, through the investigation of how genetic variations affect both pharmacokinetics and pharmacodynamics, can provide determinants of insufficient drug efficacy, side effect variation, and drug-to-drug interactions [25]. In fact, the knowledge of polymorphisms in CYP3A4, CYP2D6, and CYP2C9 genes play an essential role in the adjustment of drug dosages and in the selection of the right medicines to avoid the occurrence of side effects in cardiovascular patients [26]. To illustrate, clopidogrel is a prodrug that is generally administered as a part of dual antiplatelet therapy in acute coronary syndrome after percutaneous coronary intervention and its conversion to the active form is mainly done by CYP2C19 [27]. On the other hand, variations in the CYP2C19 gene, for example, the CYP2C19*2 allele, which causes loss of function, are associated with a reduced generation of the active metabolite that leads to decreased antiplatelet activity and increased thrombotic risk in genetically variant individuals [28,29]. Moreover, the variation in the polymorphisms in CYP2C19 among different ethnic groups may cause different drug responses in diverse populations; thus, there is a need for population-specific pharmacogenomic advisory guidelines [30]. Likewise, differences in the genes CYP2C9 and CYP2C19 affect the metabolism of other essential cardiovascular drugs such as warfarin, a situation that needs to be monitored closely to prevent side effects like bleeding or thromboembolism [31]. Cardiovascular diseases being one of the major causes of death, the use of pharmacogenomics is very necessary in the case of treatment personalizing and reduction in drug side effects [7]. Hence, pharmacogenomic tests for these major enzymes give doctors the opportunity to make dose changes or decide on a different drug treatment in advance, thus patient safety and treatment effectiveness are elevated [32,33].

This study was designed as a descriptive observational pharmacogenomic analysis. The primary aim was to determine the prevalence of CYP2D6, CYP2C19, and CYP3A4 metabolizer phenotypes in cardiac patients presenting to the emergency department. In the context of pharmacogenomics, predicted functional relevance based on CPIC refers to genetic and phenotypic variations that are known to influence drug exposure, therapeutic response, or adverse drug reaction risk. Accordingly, the primary objective of this study was to provide a descriptive analysis of CYP2D6, CYP2C19, and CYP3A4 metabolizer phenotypes in a cardiac emergency population. Structural and in silico analyses were performed as exploratory approaches to contextualize observed genetic variability and were not intended to establish functional or clinical causality. In this study, predicted functional relevance based on CPIC was defined as the presence of metabolizer phenotypes that deviate from normal enzyme activity and are associated with actionable drug–gene interactions based on CPIC recommendations. The secondary aim was to evaluate the distribution of these metabolizer phenotypes across major cardiovascular diagnostic categories. By defining these endpoints, the study seeks to provide population-level pharmacogenomic data relevant for genotype-informed cardiovascular drug therapy.

## 2. Results

**Figure 1:**
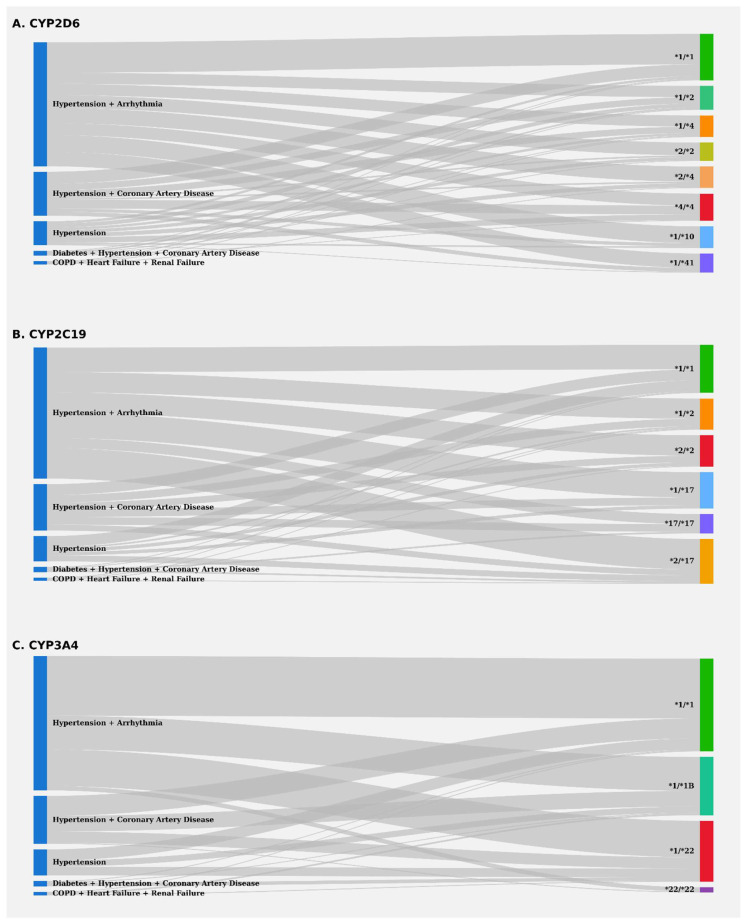
Sankey diagrams showing the distribution of genotype groups across diagnostic categories in the study cohort. (**A**) CYP2D6 genotype distribution. (**B**) CYP2C19 genotype distribution. (**C**) CYP3A4 genotype distribution. Flow width is proportional to the number of patients in each diagnosis–genotype combination. Abbreviations: COPD, Chronic Obstructive Pulmonary Disease; HF, Heart Failure; RF, Renal Failure; DM, Diabetes Mellitus; HT, Hypertension; ARR, Arrhythmia; CAD, Coronary Artery Disease.

A total of 250 patients were included in the analysis. Genotype and phenotype distributions for CYP2D6, CYP2C19, and CYP3A4 are summarized in Table 1, Table 2, Table 3, Table 4, Table 5 and Table 6, and their distribution across diagnostic categories is illustrated in Figure 1.

### 2.1. CYP2D6 Genotype and Phenotype Distribution

The distribution of CYP2D6 genotypes is presented in Table 1. The most frequent genotype was wild-type (*wt/wt*, 23.2%), followed by *4/*4 (13.6%) and *wt/*2 (12.0%). Several intermediate-function genotypes, including *wt/*4, *wt/*10, *2/*4, and *wt/*41, were also commonly observed. Phenotype distribution is summarized in Table 2. Normal metabolizers constituted the largest group (44.4%), followed closely by intermediate metabolizers (42.0%), while poor metabolizers accounted for 13.6% of the cohort. Overall, non-normal metabolizer phenotypes were identified in 55.6% of individuals.

### 2.2. CYP2C19 Genotype and Phenotype Distribution

CYP2C19 genotype distribution is shown in Table 3. The most common genotype was *wt/wt* (22.8%), followed by *2/*17 (21.2%) and *wt/*17 (17.2%). Loss-of-function (*2*) and gain-of-function (*17*) alleles were both frequently detected. Phenotype distribution (Table 4) demonstrated that intermediate metabolizers were the most prevalent group (36.0%), followed by normal (22.8%), rapid (17.2%), poor (14.8%), and ultra-rapid metabolizers (9.2%). Overall, non-normal metabolizer phenotypes were observed in 84.4% of the cohort, representing the highest prevalence among the genes investigated.

### 2.3. CYP3A4 Genotype and Activity Distribution

The distribution of CYP3A4 genotypes is presented in Table 5. The majority of individuals carried the *wt/wt* genotype (42.8%), followed by *wt/*22 (28.0%) and *wt/*1B (26.8%), while *22/*22 was rare (2.4%). Phenotype-based activity grouping (Table 6) showed that normal activity was observed in 69.6% of individuals, while decreased activity was present in 30.4% of the cohort. No increased-function phenotype was identified.

### 2.4. Comparison of Metabolizer Phenotypes Across Genes

The highest prevalence of non-normal metabolizer phenotypes was observed for CYP2C19 (84.4%), followed by CYP2D6 (55.6%). CYP3A4 exhibited a lower proportion of altered activity (30.4%), with most individuals classified as normal metabolizers.

### 2.5. Distribution Across Diagnostic Categories

The distribution of genotype groups across major cardiovascular diagnostic categories is illustrated in Figure 1. Sankey diagrams demonstrate heterogeneous but overlapping genotype distributions across diagnoses, with no single genotype restricted to a specific clinical category. While numerical differences were observed between diagnostic groups, these findings reflect descriptive patterns and should be interpreted cautiously given the non-inferential design of the study.

### 2.6. Hardy–Weinberg Equilibrium Analysis

Hardy–Weinberg equilibrium (HWE) analysis demonstrated statistically significant deviations for all investigated genes, including CYP2D6 (χ^2^ = 94.71, *p* < 0.001), CYP2C19 (χ^2^ = 39.12, *p* < 0.001), and CYP3A4 (χ^2^ = 13.87, *p* < 0.001). These deviations may reflect the non-random structure of the study population, which consists of patients with specific cardiovascular conditions rather than a representative general population sample. Additionally, factors such as selection bias, population stratification, or genotyping-related technical considerations may have contributed to the observed departures from equilibrium. Therefore, the HWE results should be interpreted within the clinical and methodological context of the study.

**Figure 2:**
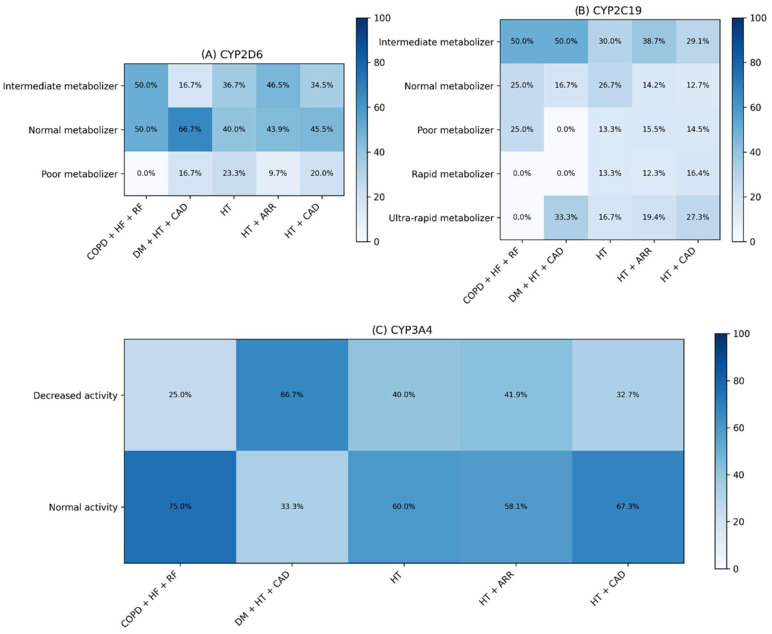
Heatmap visualization of metabolizer phenotype distributions across clinical diagnostic categories. Abbreviations: COPD, Chronic Obstructive Pulmonary Disease; HF, Heart Failure; RF, Renal Failure; DM, Diabetes Mellitus; HT, Hypertension; ARR, Arrhythmia; CAD, Coronary Artery Disease. (**A**) CYP2D6, (**B**) CYP2C19, and (**C**) CYP3A4. Each cell represents the percentage of patients within a given diagnostic group classified into the corresponding metabolizer phenotype or enzyme activity category, calculated relative to the total number of individuals in that subgroup. Color intensity reflects the relative proportion of each phenotype. CYP2C19 exhibited the highest variability and the greatest prevalence of non-normal metabolizer phenotypes, whereas CYP3A4 activity remained relatively stable across diagnostic categories with only modest variation. CYP2D6 demonstrated a balanced distribution of normal and intermediate metabolizers across most conditions.

Heatmap visualization of metabolizer phenotype distributions across diagnostic categories is presented in Figure 2. The figure illustrates the proportion of patients within each clinical subgroup classified according to CYP2D6, CYP2C19, and CYP3A4 metabolizer phenotypes or activity levels. For CYP2D6 (Figure 2A), normal and intermediate metabolizers were distributed across all diagnostic categories with comparable proportions, while poor metabolizers represented a smaller subgroup without clear clustering. For CYP2C19 (Figure 2B), phenotype distributions showed greater variability. Intermediate metabolizers predominated across most groups, while rapid and ultra-rapid metabolizers were relatively more frequent in coronary artery disease and diabetes subgroups. For CYP3A4 (Figure 2C), enzyme activity distribution appeared relatively stable across diagnostic categories, with normal activity predominating and decreased activity observed at moderate proportions. Overall, the heatmap demonstrates heterogeneous but overlapping phenotype distributions across diagnostic groups.

## 3. Discussion

Importantly, clinical interpretation in pharmacogenomics relies on functional metabolizer phenotypes rather than the mere presence of variant alleles. Accordingly, this study emphasizes phenotype-based classification to ensure clinical relevance and consistency with established guidelines. Rather than testing a predefined therapeutic hypothesis, this study aimed to provide a comprehensive descriptive overview of clinically relevant pharmacogenomic variation in an emergency cardiac population, thereby establishing a foundation for future genotype–outcome association studies. Due to the intricacy of genetic variations and their influence on drug metabolism, careful methods are required for proper identification and application in the clinic. One approach to improving pharmacogenomic interpretation is the use of comprehensive genotyping strategies that consider not only common single-nucleotide polymorphisms but also structural variants and copy number variation, particularly for highly polymorphic genes such as CYP2D6. In the present study, however, phenotype assignment was based solely on directly genotyped alleles, as copy number variation analysis was not performed [4]. Moreover, experiments need to consider the variability of different ethnic groups and regions in terms of allele frequencies. This is because such differences may have a considerable impact on how broadly pharmacogenomic results can be applied [34]. Besides that, phenotyping experiments, for example, those that use probe drugs, can offer a very helpful functional confirmation of genotype-based metabolism capacity prediction when evaluating complicated metabolizer phenotypes [35].

Our study contributes novel data on CYP2D6, CYP2C19, and CYP3A4 phenotypic variability in a cardiac emergency population, demonstrating a remarkably high prevalence of non-normal metabolizer phenotypes with predicted functional relevance based on CPIC. The highest prevalence of non-normal metabolizer phenotypes was observed in CYP2C19 (84.4%), followed by CYP2D6 (55.6%), while CYP3A4 exhibited a lower proportion of altered activity (30.4%), with normal activity predominating. These findings highlight substantial interindividual differences in metabolic capacity among patients presenting with acute cardiovascular conditions.

CYP2D6 phenotypes showed that intermediate metabolizers were predominant across most clinical diagnoses, particularly in hypertension and arrhythmia. Given the established role of CYP2D6 in the metabolism of commonly used β-blockers, variability in CYP2D6 activity may influence interindividual differences in drug exposure. However, no pharmacokinetic or treatment response analyses were performed in this study. These findings are consistent with previous reports demonstrating that CYP2D6 genetic variability affects β-blocker pharmacokinetics [36,37]. CYP2C19 phenotype distributions varied considerably across diagnoses. Ultra-rapid metabolizers were especially common among diabetic and coronary artery disease patients, while poor metabolizers constituted 13–25% of all groups. These findings are consistent with studies showing that both loss-of-function and gain-of-function alleles significantly affect clopidogrel responsiveness and cardiovascular outcomes [38,39]. Although CYP3A4 activity appeared relatively stable across diagnoses, decreased activity was markedly higher in diabetic patients. Given the reliance on CYP3A4 for atorvastatin, simvastatin, diltiazem, and amlodipine metabolism, reduced activity may predispose these patients to elevated plasma drug concentrations and adverse effects.

The co-occurrence of polymorphisms across multiple CYP450 genes in a large proportion of patients highlights the complexity of pharmacogenetic influence in acute cardiovascular care. Multi-gene involvement is particularly important because emergency therapy often includes combinations of CYP2D6, CYP2C19, and CYP3A4 substrate drugs. These findings support integrating pharmacogenomic testing into routine emergency cardiovascular management. Such functional measurements are vital to link genetic differences to actual drug metabolism rates; thus, they represent an important step towards understanding the phenotypic expression of the patients’ genotypes in diverse populations. By employing these sophisticated genotyping and phenotyping techniques, the cardiology field will be able to craft sound, practically proven, guideline-based approaches for genotype-driven drug therapy, thus leaving behind the stage of general therapeutic recommendations and entering that of highly personalized treatment regimens [14]. As an example, different ethnic groups may differ in the presence of certain CYP2D6 alleles, with Caucasians being more likely to have the wild-type allele, whereas CYP2D6*10 is prevalent in Asians; thus, the metabolic capacities for a great number of cardiovascular drugs may be different in these two populations [40].

Therefore, a deep understanding of these allele distributions specific to populations is a must for proper risk stratification and dose adjustments in clinical practice [30]. In addition, the genetic polymorphisms of the CYP450 gene family, especially the CYP3A4, CYP2D6, and CYP2C9 members, are the major factors that determine the individual differences in drug metabolism. As a result, a cardiovascular patient may suffer from adverse drug reactions or receive inadequate therapeutic efficacy [41]. Such variations compel a personalized treatment plan, especially when drugs with a narrow therapeutic window are used to effectively treat the patient and at the same time reduce the risk of therapy-related complications. Advanced genotyping methods, for instance, next-generation sequencing, can evaluate the polymorphic genes more thoroughly and detect both common and rare variants that local targeted genotyping panels might not be able to identify [42]. Such a comprehensive strategy makes it possible to pinpoint the drug response as well as the risk of an adverse event more accurately, thus giving the doctor the opportunity to adjust the treatment plan in the most suitable way for the individual cardiac patient [10]. Artificial intelligence and machine learning approaches have been proposed as future tools for integrating complex pharmacogenomic, clinical, and demographic data to support decision-making in cardiovascular therapy [43].

Cardiology is a field where this personalized medicine method is extremely important, as adverse drug reactions to cardiovascular medications continue to be a significant issue, although drug treatments have been improved [19]. The use of pharmacogenomic testing in everyday medical practice, especially for genes like CYP2C19 to select antiplatelet therapy after percutaneous coronary intervention, is a clear example of the healthcare system turning to genotype-guided cardiovascular therapies as a new standard [8]. Changes in CYP2C19 have been a major factor in the research of clopidogrel’s effects, as the studies have shown that one or more loss-of-function alleles can significantly increase the risk of coronary stent thrombosis and major adverse cardiovascular events in patients who undergo percutaneous coronary intervention [22]. Consequently, those who are at high risk should have their antiplatelet regimens optimized by quick and precise CYP2C19 genotyping [16].

Pharmacogenomic revelations regarding CYP2C19 are a major factor that changes the way doctors select antiplatelet agents, different from the standard therapy or the dosage of the medication to be given, to reduce the risk and increase therapeutic efficacy in cardiovascular patients [24,44]. For example, pre-testing CYP2C19 genotyping would be the most effective way to prevent the occurrence of cardiovascular disease side effects by identifying patients who will not respond to clopidogrel, allowing the selection of more potent antiplatelet agents such as prasugrel or ticagrelor [13]. Apart from that, it has been found that advanced sequencing methods like long-read sequencing have a crucial role in unravelling complex genes such as CYP2C19 and giving a much more accurate phenotype prediction than the traditional methods [45]. The detailed genetic information allows for a more accurate and personalized way of choosing the drug and determining its dose, thereby making the use of cardiovascular drugs safer and more effective [17].

Phenotyping methods are essential; they have been widely used to assess the functional effects of CYP450 polymorphisms by directly measuring enzyme activity, through the administration of specific probe drugs. In this way, they provide a real-time, in vivo assessment of the metabolic capacity of an individual. These functional tests might be extremely helpful, especially in situations where the genetic variations cannot explain drug responses fully; thus, they reveal a complete picture of the individual’s metabolism for personalized therapeutic adjustments. It is especially important for cardiac patients when accurate drug dosing is needed to prevent adverse events or therapeutic failure. As an illustration, the case of clopidogrel bioactivation by CYP2C19 is a clear example where the genetic variations like CYP2C19*2 loss-of-function allele lead to decreased active metabolite formation and therefore clinical outcomes. Hence, treatment changes based on genotype are required to avoid cardiovascular risks [46].

Human carriers of loss-of-function alleles for CYP2C19 have a lower ability to metabolize clopidogrel. As a result, they have less platelet inhibition and a higher chance of thrombotic events after percutaneous coronary intervention [32]. Therefore, according to population-specific pharmacogenomic evidence that may inform therapeutic decision-making that have been updated now use CYP2C19 genotype to guide antiplatelet therapy. They suggest other P2Y12 inhibitors for intermediate and poor metabolizers to allow therapeutic outcomes to be as expected and avoid cardiovascular and cerebrovascular side effects such as stroke [26]. The presence of polymorphisms in the CYP2C19 gene has significant clinical effects that go beyond the use of clopidogrel. These changes affect the metabolism of other essential cardiovascular drugs and highlight the general need for pharmacogenomic testing in cardiology [27,45]. Similarly, CYP2D6 genetic variability has been shown to influence the pharmacokinetics of metoprolol. These findings have prompted investigation into genotype-guided management strategies in specific clinical settings, including cardiac surgery. While prior studies suggest that CYP2D6 genotype may influence β-blocker exposure and response, the present study did not evaluate postoperative outcomes or atrial fibrillation risk. Therefore, any potential clinical impact should be interpreted considering existing literature rather than inferred from our data [47].

Though pharmacogenomic testing has the utility and the evidence in its favor, a number of issues are impeding its clinical translation on a large scale, among which are the difficulty of integrating genetic data into standard clinical workflows and the necessity of having uniform guidelines in different healthcare systems. In addition, the differences in the styles of clinical notes among various healthcare providers and institutions significantly increase the noise, thus making it very difficult to get accurate phenotypic data for pharmacogenomic research and implementation from those notes [29]. Besides that, economic factors like the issue of cost-effectiveness of routine testing and the matter of reimbursement, making it hard to be adopted in a widespread manner, are behind the wall of pharmacogenomics [17,41]. The way out of these problems should include, among other things, well-functioning clinical decision support systems, broad educational programs for healthcare providers, and the collaborative efforts of healthcare providers to both establish and implement harmonized pharmacogenomic guidelines that are evidence-based and clinically actionable [48]. Comprehensive research will help in understanding the pharmacogenetics of different populations, especially with respect to the drugs that are used in acute emergencies [49]. Besides that, extending the cardiology-based pharmacogenomics research to the inclusion of those populations that have been ignored so far is very important if one is to guarantee that precision medicine is implemented fairly and effectively in cardiology.

The high proportion of individuals with polymorphisms in multiple CYP450 enzymes reflects a pattern with predicted functional relevance based on CPIC: many cardiac patients simultaneously carry metabolic alterations affecting several drug classes. This multi-gene variability is especially relevant in emergency settings, where rapid pharmacologic intervention is common and drug-gene interactions can directly influence outcomes. Our findings reveal a high prevalence of CYP450 metabolizer phenotypes with predicted functional relevance based on CPIC in emergency cardiac patients and demonstrate variability across clinical diagnostic categories. These results provide population-level pharmacogenomic data that may inform future investigations evaluating the role of genotype-guided strategies in cardiovascular therapy, including antiplatelets, β-blockers, statins, and calcium channel blockers. Finally, considering this is a hospital-based study utilizing clinical and genetic data, future research could benefit from an integrated study design that explicitly links clinical data—including treatment, outcomes, and demographic information—with the genotypic results. Such an approach would enable more comprehensive genotype–phenotype correlation analyses and facilitate translation into personalized therapeutic strategies. However, prospective outcome-based studies are required before routine clinical implementation can be recommended.

Several limitations should be acknowledged. First, this was a retrospective descriptive study without pharmacokinetic measurements, clinical outcome evaluation, or inferential hypothesis testing. Second, CYP2D6 CNV analysis was not performed, limiting full characterization of duplication-associated phenotypes. Third, subgroup analyses across diagnostic categories were based on variable and sometimes small sample sizes. Fourth, the work lacks information on the medical treatment of patients, which is critical for pharmacogenetic research due to the possibility of phenoconversion, in which CYP inhibitors or inducers can change the phenotype. While this phenomenon is acknowledged in the context of cardiovascular therapies [46], data regarding whether patients were taking inhibitors or inducers at the time of blood collection were not available in this cohort. Accordingly, the findings should be interpreted as population-level descriptive data that may inform future prospective investigations.

## 4. Materials and Methods

For the study, 250 patients who presented to the Emergency Department of Pamukkale University Faculty of Medicine Hospital received a diagnosis of cardiovascular disease in the emergency setting and were scheduled for diagnostic evaluation and treatment for this reason, were included. This retrospective descriptive observational pharmacogenomic study was conducted at the Emergency Department of Pamukkale University Faculty of Medicine Hospital. Medical records and stored blood samples of 250 patients diagnosed with cardiovascular disease between January 2023 and December 2024 were reviewed.

The primary endpoint was the prevalence of CYP2D6, CYP2C19, and CYP3A4 metabolizer phenotypes. Secondary endpoints included the distribution of these phenotypes across major cardiovascular diagnostic categories [hypertension, arrhythmia, coronary artery disease, diabetes, heart failure, COPD, and renal failure].

This study was conducted in accordance with the Declaration of Helsinki and its later amendments. Ethical approval was obtained from the Pamukkale University Faculty of Medicine Clinical Research Ethics Committee (Approval No: E-60116787-020-782742; Date of approval: 19 November 2025). Written informed consent had been obtained from all participants at the time of sample collection. All data were anonymized prior to analysis.

The case report form includes sociodemographic information (age, sex, educational level), clinical data, symptom onset time, vital signs, physical examination findings, medical history (hypertension, diabetes, coronary artery disease, atrial fibrillation) history of hospitalization for a similar diagnosis, smoking/alcohol use, and the presence of coronary artery disease in first-degree relatives for patients who presented to the emergency department, received a cardiovascular disease diagnosis, and provided informed consent to participate in the study. A total of 250 individuals were included in this pharmacogenetic study. Peripheral venous blood samples (2 mL) were collected into EDTA tubes and stored at −20 °C until analysis.

### 4.1. DNA Extraction and Genotyping

Genomic DNA was purified from whole blood using the QIAamp DNA Blood Mini Kit (Qiagen, Hilden, Germany) according to the manufacturer’s protocol. DNA concentration and purity were assessed using a NanoDrop spectrophotometer (Thermo Fisher Scientific, Waltham, MA, USA). Samples with an A260/A280 ratio between 1.8 and 2.0 were considered acceptable, and DNA concentrations were normalized to 20–50 ng/µL prior to analysis.

Genotyping was performed using validated TaqMan allelic discrimination assays (Thermo Fisher Scientific, USA) on an Applied Biosystems 7500 Real-Time PCR System. The CYP2D6 alleles analyzed included *2, *4 (1846G>A), *10, and *41. In addition, CYP2C19*2 (681G>A), CYP2C19*17 (−806C>T), CYP3A4*22 (intron *6* C>T), and CYP3A4*1B (−392A>G) variants were assessed. Allelic discrimination was carried out using FAM (variant allele) and VIC (reference allele) fluorescence channels in accordance with the manufacturer’s instructions. Genotype calling was performed using Applied Biosystems SDS Software (version 2.3). Genotyping call rates exceeded 98% for all analyzed variants. As a quality control measure, 10% of samples were randomly selected for repeat genotyping, and complete concordance was observed.

These variants have predicted functional relevance based on CPIC in drug metabolism and have been chosen based on previous pharmacogenetic-related studies [50,51,52]. Results were cross-checked with the Clinical Pharmacogenetics Implementation Consortium (CPIC) guidelines to determine the corresponding metabolizer phenotype [e.g., CYP2D6 *4/*4 → poor metabolizer; CYP2C19 *17/*17 → ultrarapid metabolizer] [53]. Genetic variation was assessed at the allele level, while clinical interpretation was based on CPIC-defined metabolizer phenotypes. Individuals were not classified as ‘polymorphic’ or ‘non-polymorphic’; instead, phenotype categories reflecting functional enzyme activity were used. Predicted functional relevance based on CPIC was operationalized as the identification of CYP2D6, CYP2C19, and CYP3A4 metabolizer phenotypes that are associated with altered drug metabolism and for which therapeutic recommendations exist in CPIC guidelines. Individuals classified as poor, intermediate, rapid, or ultra-rapid metabolizers were considered to carry pharmacogenomic variation with predicted functional relevance based on CPIC. Wild-type homozygotes were classified as normal metabolizers, consistent with CPIC definitions. For CYP2D6, phenotype classification followed CPIC activity score criteria, with *2 considered a fully functional allele. Only directly genotyped alleles were used in phenotype assignments. Copy-number variation was not assessed in this study.

### 4.2. Statistical Analysis

This study was designed as a retrospective descriptive pharmacogenomic prevalence analysis. No hypothesis-driven inferential comparisons were prespecified. Categorical variables are presented as counts and percentages. Overall prevalence estimates for metabolizer phenotypes were calculated using the total cohort size (*n* = 250) as the denominator for each gene. For analysis stratified by clinical diagnosis, percentages were calculated using subgroup-specific denominators corresponding to the number of individuals within each diagnostic category. To provide measures of statistical uncertainty appropriate for prevalence studies, 95% confidence intervals (CI) were calculated for primary phenotype prevalence estimates using binomial exact methods.

Genotype-to-phenotype assignment followed CPIC activity score definitions and was limited strictly to directly genotyped alleles. Copy-number variation was not assessed; therefore, duplication-dependent ultra-rapid metabolizer status was not assigned. Quality control procedures ensured genotype call rates > 98%. Samples with ambiguous amplification curves were repeated. No indeterminate or unresolved genotype calls were included in the final analysis dataset. All analyses were performed using SPSS Statistics version 26.0 (IBM Corp., Armonk, NY, USA).

## 5. Conclusions

This descriptive pharmacogenomic study demonstrates a high prevalence of metabolizer phenotypes with predicted functional relevance based on CPIC among cardiac patients presenting to the emergency department. Non-normal metabolizer phenotypes were identified in 55.6% of individuals for CYP2D6, 84.4% for CYP2C19, and 30.4% for CYP3A4, reflecting substantial interindividual variability in drug metabolism capacity within this population.

A considerable proportion of patients exhibited altered metabolizer phenotypes in more than one CYP450 enzyme, highlighting the potential complexity of multi-gene pharmacogenomic variability in acute cardiovascular care. Intermediate metabolizer phenotypes predominated for both CYP2D6 and CYP2C19 across most clinical diagnoses, while CYP3A4 activity showed relatively stable distribution with modest variation between diagnostic categories. These findings provide population-level pharmacogenomic data that may inform future prospective, outcome-based studies evaluating genotype-guided cardiovascular therapy.

## Figures and Tables

**Table 1:** Distribution of CYP2D6 genotypes and predicted metabolizer phenotypes in the study population [*n* = 250].

Genotype	*n*	%	Predicted Phenotype
*wt/wt*	58	23.2	Normal metabolizer
*4/*4	34	13.6	Poor metabolizer
wt/*2	30	12.0	Normal metabolizer
wt/*4	27	10.8	Intermediate metabolizer
*wt/wt0*	27	10.8	Intermediate metabolizer
*2/*4	27	10.8	Intermediate metabolizer
wt/*41	24	9.6	Intermediate metabolizer
*2/*2	23	9.2	Normal metabolizer

Abbreviations: *wt,* wild-type.

**Table 2:** Distribution of predicted CYP2D6 metabolizer phenotypes in the study population [*n* = 250].

Predicted Phenotype	*n*	%
Normal metabolizer	111	44.4
Intermediate metabolizer	105	42.0
Poor metabolizer	34	13.6

**Table 3:** Distribution of CYP2C19 genotypes and predicted metabolizer phenotypes in the study population [*n* = 250].

Genotype	*n*	%	Predicted Phenotype
*wt/wt*	57	22.8	Normal metabolizer
*2/*17	53	21.2	Intermediate metabolizer
wt/*17	43	17.2	Rapid metabolizer
wt/*2	37	14.8	Intermediate metabolizer
*2/*2	37	14.8	Poor metabolizer
*17/*17	23	9.2	Ultra-rapid metabolizer

Abbreviations: *wt*, wild-type.

**Table 4:** Distribution of predicted CYP2C19 metabolizer phenotypes in the study population [*n* = 250].

Predicted Phenotype	*n*	%
Intermediate metabolizer	90	36.0
Normal metabolizer	57	22.8
Rapid metabolizer	43	17.2
Poor metabolizer	37	14.8
Ultra-rapid metabolizer	23	9.2

Abbreviations: *wt*, wild-type.

**Table 5:** Distribution of CYP3A4 genotypes and predicted activity groups derived from genotype in the study population [*n* = 250].

Genotype	*n*	%	Predicted Activity
*wt/wt*	107	42.8	Normal activity
wt/*22	70	28.0	Decreased activity
wt/*1B	67	26.8	Normal activity
*22/*22	6	2.4	Decreased activity

Abbreviations: *wt*, wild-type.

**Table 6:** Corrected distribution of predicted CYP3A4 activity groups derived from genotype in the study population [*n* = 250].

Predicted Activity	*n*	%
Normal activity	174	69.6
Decreased activity	76	30.4

Abbreviations: *wt*, wild-type.

## Data Availability

The original contributions presented in this study are included in the article. Further inquiries can be directed to the corresponding author(s).

## Abbreviations

The following abbreviations are used in this manuscript:

CYP450	Cytochrome P450
CPIC	Clinical Pharmacogenetics Implementation Consortium
CAD	Coronary artery disease
ACS	Acute coronary syndrome
COPD	Chronic obstructive pulmonary disease
HWE	Hardy–Weinberg equilibrium
CNV	Copy-number variation
EDTA	Ethylenediaminetetraacetic acid
PCR	Polymerase chain reaction
SNP	Single-nucleotide polymorphism
*wt*	Wild-type
FAM/VIC	Fluorescent dye markers for TaqMan assays
CI	Confidence interval
